# The Study of the Association of Polymorphisms in *LSP1*, *GPNMB*, *PDPN*, *TAGLN*, *TSPO*, and *TUBB6* Genes with the Risk and Outcome of Ischemic Stroke in the Russian Population

**DOI:** 10.3390/ijms24076831

**Published:** 2023-04-06

**Authors:** Andrey V. Khrunin, Gennady V. Khvorykh, Anna S. Arapova, Anna E. Kulinskaya, Evgeniya A. Koltsova, Elizaveta A. Petrova, Ekaterina I. Kimelfeld, Svetlana A. Limborska

**Affiliations:** 1 National Research Centre “Kurchatov Institute”, Kurchatov Sq. 2, Moscow 123182, Russia; 2 Faculty of Biotechnology and Industrial Ecology, Mendeleev University of Chemical Technology of Russia, Miusskaya Sq. 9, Moscow 125047, Russia; 3 Department of Neurology, Neurosurgery and Medical Genetics of Pirogov Russian National Research Medical University, Moscow 117997, Russia

**Keywords:** single nucleotide polymorphisms, ischemic stroke, ischemic stroke outcome, rat model of brain ischemia

## Abstract

To date, there has been great progress in understanding the genetic basis of ischemic stroke (IS); however, several aspects of the condition remain underexplored, including the influence of genetic factors on post-stroke outcomes and the identification of causative loci. We proposed that an analysis of the results obtained from animal models of brain ischemia could be helpful. To this end, we developed a bioinformatic approach for exploring single-nucleotide polymorphisms (SNPs) in human orthologs of rat genes expressed differentially after induced brain ischemia. Using this approach, we identified and analyzed 11 SNPs from 6 genes in 553 Russian individuals (331 patients with IS and 222 controls). We assessed the association of SNPs with the risk of IS and IS outcomes. We found that the SNPs rs858239 (*GPNMB*), rs907611 (*LSP1*), and rs494356 (*TAGLN*) were associated with different parameters of IS functional outcomes. In addition, the SNP rs1261025 (*PDPN*) was associated significantly with IS itself (*p* = 0.0188, recessive model). All these associations were demonstrated for the first time. Analysis of the literature suggests that they should be characterized as being inflammation related. This supports the pivotal role of inflammation in both the incidence of stroke and post-stroke outcomes. We believe the findings reported here will help with stroke prognosis in the future.

## 1. Introduction

Stroke, including ischemic stroke (IS), is either the second- or third-most common cause of human mortality and disability worldwide [1]. Given the high global cost of stroke, even small improvements in prophylactics and prognoses could result in major positive impacts on health and treatment costs. Stroke is known to be due to modifiable risk factors [2] and is, therefore, preventable to a large extent. However, genetic factors are also known to be involved in the stroke susceptibility of individuals. With regard to subtypes of IS, estimates of heritability include 16.1% for small vessel stroke, 32.6% for cardioembolic stroke, and 40.3% for large vessel stroke [3]. The largest genome-wide association (GWA) study yet conducted revealed 89 stroke-risk loci, 61 of which were novel [4]. The outcomes of IS are also influenced by different factors, including genetic ones [5,6]. However, and in contrast to IS itself, the roles played by genetic factors have not yet been properly estimated, and our knowledge of the impact of genetic factors on post-stroke outcomes is still very limited. To date, only a few GWA studies have been carried out. These found that several loci were associated with IS outcomes [7,8,9]. However, although GWA studies have demonstrated their effectiveness in discovering trait-related loci, and are expected to be used in the future, such studies suffer from certain limitations. The most notable of these is their poor precision in identifying real causal variants or genes caused by linkage disequilibrium between the loci tested. This imprecision hinders the development of biological insights and the clinical implementation of GWA findings and also necessitates the functional validation of most of the gene associations discovered [10]. Ultimately, this deficiency highlights the need for the development of new approaches that can identify causal genetic variants and help to clarify the biological mechanisms that underlie the associations observed. Such methods might involve the enhanced downstream analysis of identified loci through the involvement of different “omics” data and advanced analytic techniques or the direct functional dissection of particular variants and genes, either in vitro or in vivo [10,11,12].

In parallel, genes involved in IS have also been searched for and characterized using animal models [13,14]. Through these, a general picture of IS pathophysiology has been developed [14]. Some of the results obtained in rodent models of stroke have been valuable for explaining associations found in humans, including GWA-assessed outcomes after IS [7], but their translation remains generally low. This is because of substantial physiological differences between model animals and humans, as well as the inadequate applicability of animal models of stroke to the real human disease due to the heterogeneity of human stroke and the use of young animals to model an aging-associated condition, amongst other issues [14]. For this reason, the creation of more human-like animal models is now being actively pursued as one means of improving translation [15]. An alternative approach is based on the belief that the “humanization” of animal models is possible only to a limited extent and that greater success is more likely to be obtained by “humanizing” the methods used for data processing, particularly, the careful selection and validation of cross-species multiomic data [16,17]. Following this approach, we developed a bioinformatics protocol with the aim of identifying genomic markers that could affect IS outcomes [18]. This involves the exploration of genomic variations in human orthologs of rat genes, which substantially change their expression in the brain in response to transient middle cerebral artery occlusion (tMCAO). We recently demonstrated the efficacy of this method in a pilot study [19]. In this paper, we present the results obtained using a slightly improved version for the validation of a new set of genes, which were differentially expressed in rats after induced brain ischemia.

## 2. Results

A total of 331 IS patients were analyzed. One patient was excluded from the analysis because he died eight days after IS. In Study 1, 69 patients were classified as the functional recovery group (modified Rankin Scale (mRS) scores of 0–1), and 261 patients were in the nonfunctional recovery group (mRS scores of 2–6). In Study 2, the functional recovery group comprised 116 patients (mRS scores of 0–2), while the nonfunctional recovery group comprised 214 patients (mRS scores of 3–6). In Study 3, the functional and nonfunctional recovery groups consisted of 175 patients (mRS scores of 0–3) and 155 patients (mRS scores of 4–6), respectively. The group of patients with an mRS score of 3 contained 59 individuals. Positive changes in the mRS scores (∆mRS > 0) were observed in 155 patients; negative changes were found in 67 patients (∆mRS < 0); and the scores of 108 patients remained stable (∆mRS = 0).

The genotype frequencies of eleven SNPs accessed in the four basic studies are presented in Table 1. Some of these demonstrated group-related specificities. The distributions of the SNP rs858239 genotypes differed between the functional and nonfunctional groups in Studies 1 and 2, while SNP rs907611 was correlated with the dynamics of changing patient mRS scores in Study 4 (Table 1). A comparison of the genotype distributions of both SNPs under other genetic models of inheritance (dominant, recessive, and overdominant) showed that associations related to the SNP rs858239 were attributed to the homozygous genotype AA, which occurred more frequently in the functional recovery groups (*p* = 0.007 and *p* = 0.009 for Studies 1 and 2, respectively) (Table S1). The association of SNP rs907611 with IS outcomes was correlated with a distinct genotype pattern in the group of patients with negative ∆mRS values. In particular, there was a higher frequency of genotype AA in this group (Table 1 and Table S1). Under alternative genetic models, several other SNPs also demonstrated associations with stroke outcomes, namely, SNP rs34323745, SNP rs494356, and SNP rs2089910. The SNPs rs34323745 and rs2089910 were found to tag alternative patterns of variation (groups of highly correlated SNPs) in the *GPNMB* and *LSP1* genes (Table 2 and Table S1). The SNP rs34323745 was also associated with outcomes in the analysis variant in which patients with an mRS score of 3 were considered along with patients with scores of 0–2 and 4–6. A review of the results of the genetic models revealed an association with the prevalence of a heterozygous genotype AC in patients with an mRS score of 3.

However, only three SNPs—rs858239, rs907611, and rs494356—remained associated with stroke outcomes after the appropriate logistic regression analysis was applied (Table 3). For the genotypic variables with significant parameters, we also estimated the overall effect of the SNPs on the models by means of the Wald test and residual deviance computation. The results of both tests confirmed the importance of the SNPs.

Because the genetics of IS are not yet clearly understood, the SNPs were also tested for association with the risk of IS. One SNP was found to be associated with the disease: rs1261025 (Table S1). This association was due to the homozygous genotype GG that occurred about three times more often in the stroke patients than in the controls. Among all the genetic models considered, the most pronounced differences were observed for the recessive model (*p* = 0.0188).

## 3. Discussion

The outcomes of IS vary from complete recovery to persistent severe disability or death. In the current study, we studied polymorphisms of six genes in patients who were alive after IS. To assess the factors affecting the outcomes of the disease, outcomes are commonly analyzed by dividing patients into functional and nonfunctional recovery groups based on individual mRS scores. The patients with low scores are then compared to those with high scores [7,20]. Because there is no consensus regarding optimal grouping, we also applied directional ∆mRS, as recently proposed by ourselves [19]. We also divided the patients into three groups using mRS. With regard to the latest variant, we considered the specific characteristics of all the patients with an mRS score of 3 (see Section 4 for details). Using this approach, some differences in the genotype distribution were found, but their importance for IS outcomes was not confirmed in the regression analysis.

As a result of testing, three SNPs associated with IS outcomes were identified. All of these are in different genes. SNP rs858239 is located at 79 bp upstream of the *GPNMB* gene (GRCh38). This gene encodes the transmembrane glycoprotein NMB (non-metastatic melanoma protein B), which was initially described as a regulator of tumor growth. Subsequently, its expression has been detected in many tissues, including bone, skin, kidney, and skeletal muscle, where it is involved in various cellular processes, such as cell differentiation, migration, and tissue regeneration [21]. *GPNMB* is also expressed in the normal central nervous system (CNS)—brain and spinal cord—of adult rats, but its expression is mainly associated with microglial cells, which are resident immune cells. For this reason, it has been proposed that GPNMB might also play a role in inflammatory processes in the CNS [22]. Various studies have suggested that GPNMB is neuroprotective. Transgenic mice with overexpressed *GPNMB* had a significantly decreased infarct volume after ischemia–reperfusion injury compared with wild-type controls [23]. The treatment of an astrocyte cell line and primary mouse astrocytes with GPNMB decreased the levels of inducible nitric oxide synthase, nitric oxide, reactive oxygen species, and the inflammatory cytokine IL-6 induced by a pro-inflammatory cytokine-mix treatment [24]. The overexpression of *GPNMB* protected against dopaminergic neurodegeneration in a 1-methyl-4-phenyl-1,2,3,6-tetrahydropyridine (MPTP) mouse model of Parkinson’s disease. It also reduced gliosis and prevented microglial morphological changes caused by MPTP treatment. In addition, recombinant GPNMB was found to attenuate LPS-induced inflammation in primary mouse microglia [25]. Interestingly, increased levels of GPNMB have been observed in brain samples and/or the cerebrospinal fluid of patients with neurodegenerative diseases such as amyotrophic lateral sclerosis, Parkinson’s disease, Alzheimer’s disease, and multiple sclerosis [24,26,27,28]. Taking into account the properties of GPNMB, it has been proposed that the protein is induced under these conditions to hold back inflammation and protect neurons [28]. However, an alternative view links elevated levels of GPNMB with an increased risk of diseases and disease stages [29,30]. Increased levels of GPNMB in plasma have also been linked with a higher risk of cardiovascular diseases, particularly myocardial infarction and heart failure [31].

A meta-analysis of GWAS studies that included 13,708 cases and 95,282 controls identified SNP rs199347 as one of the top risk loci for Parkinson’s disease [32]. Murthy and co-workers further explored the genomic region around this locus and found that SNP rs199347 was significantly associated with increased *GPNMB* expression in multiple brain regions, indicating that the *GPNMB* gene could potentially affect the risk of PD [33]. Another SNP from the *GPNMB* gene—SNP rs858239—has been associated with GPNMB protein levels in cerebrospinal fluid [34]. SNP rs858239 is one of the loci under investigation in the current study. It is in high LD, with the SNP rs199347 (r^2^ = 0.94–1.0 in European samples of the 1000 Genomes project) located in an intron of the *GPBNM* gene. We found that SNP rs858239 was associated with outcomes after IS. This association was correlated with genotype AA, which occurred more frequently in the groups of patients with a slight disability, or none at all, than in the severe-symptom groups. According to the Genotype-Tissue Expression (GTEx) project [https://gtexportal.org/home/ (accessed on 12 February 2023)] as well as Murthy and co-workers, these genotypes are associated with a minimal level of *GPNMB* expression compared with AG and, especially, GG genotypes. To the best of our knowledge, the current study is the first to report an SNP of *GPNMB* that is correlated with IS outcomes in humans. In the context of our emphasis on patients with an mRS score of 3, it should be also noted that these individuals did not differ from the patients with scores of 4–6 in terms of the genotype frequency of SNP rs858239. This finding suggests that combining these patients with those who had scores of 0–2 would have been of little or no value.

The second important locus identified in our work was SNP rs907611, located 2kb upstream of the *LSP1* gene (GRCh38). This gene encodes an intracellular F-actin binding protein—lymphocyte-specific protein 1—that is expressed in hematopoietic and endothelial cells. Because *LSP1* is involved in the regulation of actin cytoskeleton structural organization and dynamics, it plays an important role in leukocyte motility, transendothelial migration, and the adhesion of fibrinogen matrix proteins [35]. Initial studies with *LSP1*-deficient mice suggested a negative regulatory role of *LSP1* in inflammation. Researchers found increased infiltration of neutrophils in the inflamed tissues of model animals. These observations were supported by increased migrations of LSP1-deficient neutrophils and T-lymphocytes in response to chemical stimulus in vitro [35,36]. In contrast to leukocytes, *LSP1* deficiency in endothelial cells resulted in a profound decrease in neutrophil transmigration [37]. This effect was explained by the involvement of LSP1 in endothelial dome formation during leukocyte transmigration [38]. These differences between LSP1 from leukocytes and LSP1 from endothelial cells may be due to two specific features of the enzymes. First, LSP1 expressed by leukocytes is predominantly a cytosolic protein associated with the cytoskeleton, while LSP1 expressed by endothelial cells is mainly located in the nucleus. Second, in leukocytes, LSP1 is quickly phosphorylated by the stimulation of soluble chemoattractants such as fMLP and chemokines, while LSP1 phosphorylation in the endothelium requires ICAM-1-mediated neutrophil adhesion [39,40]. Nevertheless, it seems that endothelial cell-expressed LSP1 is crucial for the transendothelial migration of leukocytes and extravascular chemotaxis [40]. Moreover, *LSP1*-deficient neutrophils have been reported to exhibit impaired migration speed and chemotaxis directionality [41], and *LSP1*-expressing cells have been found to occur in inflamed tissues (e.g., T cells in synovial tissues) [36].

The excessive and uncontrolled infiltration of distinct immune cells into particular organs or tissues has been identified as a characteristic pathology of various chronic inflammatory diseases, such as psoriasis, ulcerative colitis, Crohn’s disease, asthma, and rheumatoid arthritis; many of these have been associated with *LSP1* [36,42,43]. Stroke has not yet been directly related to *LSP1*, but an association with *LSP1* has been demonstrated for blood pressure, which is one of the most important risk factors of stroke [44]. An increased level of *LSP1* mRNA expression in blood was described recently as a potential diagnostic biomarker for Parkinson’s disease [45]. In the current study, we identified an association between *LSP1* polymorphism at the rs906711 locus and IS outcomes. The outcomes were assessed by evaluating the direction of the changes in patients’ mRS scores between the 14th day and the day of admission (∆mRS). This was correlated with the AA genotype that occurred more frequently in patients with negative ∆mRS values. According to GTEx data, AA genotypes are associated with increased levels of LSP1 expression in brain tissues compared with AG and GG genotypes. Thus, the possession of the AA genotype can be considered a risk factor for a negative outcome after IS.

The final SNP associated with IS outcome—rs494356—is located in an intron of the *TAGLN* gene. This gene encodes the actin-binding protein transgelin, which is abundant in vascular and visceral smooth muscle, and it is an early marker of smooth muscle differentiation. The authors of [46] found that by inducing filamentous actin bundling, TAGLN facilitated the reorganization of the cytoskeleton and maintained the contractile phenotype of vascular smooth muscle cells (VSMCs) in rats. In another study, high levels of the expression of *TAGLN* inhibited VSMC proliferation and injury-induced neointimal hyperplasia in rats [47]. Contrarily, *TAGLN* expression is commonly downregulated in tumor cells in comparison with the cells of healthy tissue [48]. Lowered expression of *TAGLN* has also been observed in cardiovascular diseases, such as atherosclerosis (the atherosclerosis plaque), intracranial aneurysms, and aortic dissection [49,50,51]. The disruption of TAGLN promotes arterial inflammation through the activation of TNF-α-mediated NF-κB pathways followed by the production of reactive oxygen species [52]. Moreover, *TAGLN* expression has been found to decrease after treatment with TNF-α [53]. In turn, the overexpression of *TAGLN* was shown to attenuate TNF-α-induced inflammation in VSMCs [54]. Taken together, these research findings suggest that *TAGLN* can affect inflammatory damage in VSMCs.

GWAS studies have demonstrated associations between *TAGLN* and multiple traits [https://www.ebi.ac.uk/gwas/genes/TAGLN (accessed on 16 February 2023)], most of which are related to the lipid content in blood. This can be explained by inducing switching of the VSMC phenotype (from contractile to synthetic) with products of lipid metabolism [55]. In our study, the *TAGLN* SNP rs494356 was associated with IS outcomes, particularly ∆mRS. The association correlated with the occurrence of genotypes containing allele T. However, the frequency of minor homozygous genotypes was insufficient to test their distribution with reliable power. They were also rare in the GTEx data for brain tissue. As for the more frequent genotypes CT and CC, the GTEx data demonstrated that genotype CC was associated with a higher level of *TAGLN* expression than CT. Decreased *TAGLN* expression can contribute both to the destruction of the vascular wall and local inflammation and can, therefore, be expected to prevail in patients whose functional state deteriorates.

As mentioned above, all the SNPs under study were also tested for correlation with IS risk because of the limitation of existing data on stroke genetics, particularly its potential specificity in populations of different ancestries [56]. Our analysis was carried out on patients of Russian ethnicity. One of the SNPs studied was found to be associated with the risk of IS. The significant SNP rs1261025 is located in an intron of the *PDPN* gene. This gene encodes the small mucin-like transmembrane glycoprotein podoplanin. Experiments with podoplanin gene knockout in mice have demonstrated its crucial role in the normal development and functioning of the lung, heart, and lymphatic vascular system [57]. In addition, many studies have demonstrated the involvement of PDPN in the pathological processes in the brain, including development, angiogenesis, tumors, ischemic stroke, and other neurological disorders [58]. Studies on ischemic stroke have mostly been concerned with the role of PDPN and/or its partner C-type lectin-like receptor-2 (CLEC-2) in acute IS, ischemia–reperfusion injury, and IS outcome. CLEC-2 is an activator of platelets, and podoplanin is the only known ligand of CLEC-2 [59]. Researchers have found that (1) patients with acute IS had significantly higher levels of CLEC-2 in plasma and (2) higher CLEC-2 plasma levels were significantly associated with stroke progression and poorer prognosis at 90 days after admission, as well as an increased risk of death and vascular events in a one-year follow-up period [60,61]. These findings are supported by the results of experiments on model animals, which showed that the pretreatment of mice with anti-podoplanin antibodies significantly reduced the cerebral infarct volume, attenuated neurological deficits, decreased the levels of IL-18 and IL-1β, and alleviated microvascular thrombosis [62,63].

In our study, no association was found between *PDPN* polymorphisms and IS outcomes, but an association with IS itself was identified. As has been widely recognized, ischemic stroke is preceded by thromboinflammation, a process related to the activation of both platelets and immune cells [64]. It was previously proposed that the interaction between PDPN on activated macrophages and platelet CLEC-2 played a role in thromboinflammation under atherosclerosis conditions and, thus, contributed to the thrombotic properties of advanced atherosclerotic lesions [65]. Subsequently, it was demonstrated that *PDPN* expression could be induced in endothelial cells by vascular endothelial growth factor (VEGF)-A from superficial smooth muscle cells. Such mediated *PDPN* overexpression intensified platelet aggregation via podoplanin-CLEC-2 interaction, enhancing erosive injury and thrombus formation [66]. In our study, the association with IS was correlated with the occurrence of genotype GG at rs1261015, which was more frequent among the patients than the controls. According to the GTEx data, GG correlates with a high level of PDPN expression in arteries, which can promote enhanced thrombocyte aggregation and increased incidence of IS in individuals.

In conclusion, we continued to explore the possibility of translating the results obtained from model animals to human subjects. Using a recently developed approach, we applied the results of an analysis of gene expression in rat brain after tMCAO to search for genetic markers of IS. The associations found confirmed the efficacy of this approach. However, it should be noted that although all the loci originated from the genes correlated with cerebral ischemia–reperfusion injury, only three of them (SNPs in genes *GPNMB*, *LSP1*, and *TAGLN*) were associated with outcomes after IS, while one locus (SNP rs1261025 in the *PDPN* gene) demonstrated associations with IS itself. These differences in the SNP/gene effects can be attributed to interspecies differences between rats and humans with respect to gene functioning. Furthermore, an analysis of the literature suggested that all these should be characterized as inflammation-related, thereby supporting the pivotal role of inflammation in both the incidence of stroke and post-stroke outcomes. To understand this further and develop better methods for stroke prognosis, further studies are required. We believe that our approach could be helpful because all the associations that we identified using this method were described for the first time. However, due to technical limitations, only a portion of local eQTLs in several tens of genes have been evaluated and tested in our studies to date. For this reason, the global validation of all the genes associated with post-ischemic injury in rat brain for human subjects still needs to be completed in the future.

## 4. Materials and Methods

### 4.1. Study Subjects

The association study was carried out in a subgroup of patients with IS admitted to the Neurologic Department of Moscow City Clinical Hospital No. 31 during 2008–2011. The detailed procedures of patient enrollment and data collection have been described previously [67]. Briefly, the study group comprised men and women aged ≥ 55 years who had been diagnosed with their first IS and were taken to the hospital at the acute stage of the disease. Patients with transitory ischemic attacks, recurrent stroke, hemorrhagic stroke, acute myocardial infarction, decompensated heart failure, or other severe accompanying conditions, including autoimmune and infectious diseases, were not recruited into the study. To assess stroke severity, the National Institutes of Health Stroke Scale was used [68]. The functional outcome after the stroke was graded using a modified Rankin Scale (mRS) [69]. The severity and outcome were assessed twice—on days 1 and 14 after the stroke event. To obtain a group with homogeneous ancestry, only the patients who self-described as Russian were included. In total, 331 patients (166 males and 165 females; mean age 71.68 ± 8.69) with IS were enrolled. The control group included 222 healthy individuals (109 males and 113 females; mean age 70.20 ± 9.63) with Russian ancestry from regions of central European Russia. All the subjects provided written informed consent for participation.

### 4.2. Selection and Genotyping of Markers

DNA was isolated from peripheral blood cells by proteinase K treatment, followed by extraction with phenol–chloroform [70]. The principles of the selection of the polymorphic markers analyzed in the study were described in our recently developed protocol [18] and included the following main steps: (1) the selection of rat genes with the most significant changes in their expression in the brain in response to temporal artery occlusion; (2) the identification of the human orthologs of these rat genes; (3) the identification of SNPs in human genes and any tag SNPs among them using whole-genome sequence data from an appropriate population (i.e., CEU population from the 1000 Genomes Project); (4) the annotation and identification of functionally important tag SNPs that affect the expression of the genes studied (expression quantitative trait loci (eQTLs)) using the data of the Genotype-Tissue Expression (GTEx) project. In evaluating the tag SNPs, only those SNPs associated with changes in the expression of corresponding genes in human brain tissues were considered. The SNPs of 6 genes from the initial list of 28 genes were found to be brain-associated eQTLs. The final selection was made by comparing the results of the evaluation of the functional features of the SNPs using HaploReg [71] and RegulationSpotter [72]. In total, there were eleven such SNPs in six genes: *LSP1*, *GPNMB*, *PDPN*, *TAGLN*, *TSPO*, and *TUBB6* (Table 4).

The selected tag SNPs were genotyped with the use of self-designed TaqMan real-time PCR assays. The name of the software used, as well as the description of the components of the PCR reaction mixture and the general cycling conditions, was the same as described in our previous study [19]. The sequences of the primers and probes used in the current study are presented in Table 4.

### 4.3. Statistical Analysis

The aim of this study was to explore the associations between genetic variants and IS outcomes as well as the risk of developing the disease. First, the effects of SNPs on stroke outcomes were assessed through their associations with mRS scores. Following established dichotomous schemes, the scores were analyzed as follows: (1) mRS scores of 0–1 vs. scores of 2–6 (Study 1); (2) mRS scores of 0–2 vs. scores of 3–6 (Study 2); (3) mRS scores of 0–3 vs. scores of 4–6 (Study 3). In addition to these analyses, the patients with an mRS score of 3 were placed in a separate group and compared with the patients with mRS scores of 0–2 and 4–6. These patients differed from the patients with scores of 0–2 in that they had a moderate disability and required some help in everyday life. They also differed from the patients with scores of 4–6 because they could walk without assistance. Walking ability (leg motor ability) has been suggested to be an independent predictor of functional outcome [20] and, thus, can be considered as a criterion for dividing patients. Studies 1 to 3 were based on the mRS scores assessed on day 14. The second approach was based on an evaluation of the differences in the direction of the post-stroke outcomes [19]. These were assessed through the calculation of the individual changes (∆ values) in the mRS scores given to patients on days 1 and 14, i.e., ∆mRS = mRS1 − mRS14, and were defined as positive (∆mRS > 0), negative (∆mRS < 0), or stable (∆mRS = 0). To detail the characteristics of the particular groups, the ∆mRS values were also compared to each other individually and in combinations (∆mRS < 0 + ∆mRS = 0 vs. ∆mRS > 0; ∆mRS > 0 + ∆mRS = 0 vs. ∆mRS < 0; ∆mRS < 0 vs. ∆mRS = 0; ∆mRS > 0 vs. ∆mRS = 0; and ∆mRS < 0 vs. ∆mRS > 0). To assess the relationships between the SNPs and IS risk and outcomes, genotypic χ^2^ tests were used. To define the contribution of the particular genotypes, dominant, recessive, and overdominant genetic models were applied. Minor alleles were considered risk alleles in all the models (Table S2). To find the variables that were significantly associated with the phenotype, logistic regression was used. The factors considered were sex, age, and the eleven SNPs. When the outcome variable contained two classes, we applied binomial logistic regression; when it had three classes, we used ordinal logistic regression [73]. The significance of the associations was set at *p* < 0.05. The statistical analyses were performed using Statistica software v.8.0 (StatSoft, Inc., Tulsa, OK, USA), GraphPadInStat v.3.00 (GraphPad Software, CA, USA), and Haploview software v. 4.2 [74]. Regression analysis and evaluation of the models were carried out with the MASS, pscl, and aod R-packages [75,76].

## Figures and Tables

**Table 1:** Genotype frequency in the groups of patients from Studies 1, 2, 3, and 4.

SNP	Allele	Study 1 *		*p*	Study 2 *		*p*	Study 3 *		*p*	Study 4 *			*p*
		mRS 0–1	mRS 2–6		mRS 0–2	mRS 3–6		mRS 0–3	mRS 4–6		∆mRS > 0	∆mRS < 0	∆mRS = 0	
rs858239	A/A	0.30 (21)	0.16 (42)	0.0091	0.27 (31)	0.15 (32)	0.0331	0.22 (39)	0.15 (24)	0.2589	0.22 (34)	0.13 (9)	0.19 (20)	0.5496
	A/G	0.48 (33)	0.48 (124)		0.44 (51)	0.50 (106)		0.45 (78)	0.51 (79)		0.45 (70)	0.55 (37)	0.46 (50)	
	G/G	0.22 (15)	0.36 (95)		0.29 (34)	0.36 (76)		0.33 (58)	0.34 (52)		0.33 (51)	0.31 (21)	0.35 (38)	
rs34323745	A/A	0.01 (1)	0.03 (7)	0.1119	0.03 (3)	0.02 (5)	0.1431	0.03 (6)	0.01 (2)	0.4429	0.03 (5)	0.00 (0)	0.03 (3)	0.6606
	A/C	0.19 (13)	0.31 (80)		0.22 (25)	0.32 (68)		0.27 (48)	0.29 (45)		0.28 (43)	0.27 (18)	0.30 (32)	
	C/C	0.80 (55)	0.67 (174)		0.76 (88)	0.66 (141)		0.69 (121)	0.70 (108)		0.69 (107)	0.73 (49)	0.68 (73)	
rs11267036	G/G	0.01 (1)	0.02 (6)	0.7061	0.02 (2)	0.02 (5)	0.6852	0.02 (3)	0.03 (4)	0.8588	0.03 (4)	0.01 (1)	0.02 (2)	0.6253
	C/G	0.22 (15)	0.26 (67)		0.22 (26)	0.26 (56)		0.25 (44)	0.25 (38)		0.26 (41)	0.18 (12)	0.27 (29)	
	C/C	0.77 (53)	0.72 (188)		0.76 (88)	0.71 (153)		0.73 (128)	0.73 (113)		0.71 (110)	0.81 (54)	0.71 (77)	
rs907611	A/A	0.09 (6)	0.13 (34)	0.2044	0.10 (12)	0.13 (28)	0.1686	0.13 (22)	0.12 (18)	0.7418	0.12 (18)	0.21 (14)	0.07 (8)	0.0494
	A/G	0.52 (36)	0.41 (106)		0.50 (58)	0.39 (84)		0.45 (78)	0.41 (64)		0.45 (70)	0.43 (29)	0.40 (43)	
	G/G	0.39 (27)	0.46 (121)		0.40 (46)	0.48 (102)		0.43 (75)	0.47 (73)		0.43 (67)	0.36 (24)	0.53 (57)	
rs2089910	A/A	0.07 (5)	0.08 (21)	0.9577	0.07 (8)	0.08 (18)	0.4976	0.08 (14)	0.08 (12)	0.8730	0.08 (13)	0.06 (4)	0.08 (9)	0.2184
	A/G	0.39 (27)	0.38 (98)		0.34 (40)	0.40 (85)		0.37 (64)	0.39 (61)		0.34 (52)	0.34 (23)	0.46 (50)	
	G/G	0.54 (37)	0.54 (142)		0.59 (68)	0.52 (111)		0.55 (97)	0.53 (82)		0.58 (90)	0.60 (40)	0.45 (49)	
rs494356	T/T	0.01 (1)	0.03 (9)	0.6892	0.02 (2)	0.04 (8)	0.5108	0.03 (6)	0.03 (4)	0.9005	0.03 (4)	0.01 (1)	0.05 (5)	0.2398
	T/C	0.26 (18)	0.25 (66)		0.28 (32)	0.24 (52)		0.25 (44)	0.26 (40)		0.21 (33)	0.25 (17)	0.31 (34)	
	C/C	0.72 (50)	0.71 (186)		0.71 (82)	0.72 (154)		0.71 (125)	0.72 (111)		0.76 (118)	0.73 (49)	0.64 (69)	
rs664922	G/G	0.13 (9)	0.08 (21)	0.2555	0.12 (14)	0.07 (16)	0.3753	0.12 (21)	0.06 (9)	0.1432	0.10 (15)	0.06 (4)	0.10 (11)	0.7847
	G/T	0.30 (21)	0.39 (102)		0.35 (41)	0.38 (82)		0.35 (62)	0.39 (61)		0.35 (54)	0.42 (28)	0.38 (41)	
	T/T	0.57 (39)	0.53 (138)		0.53 (61)	0.54 (116)		0.53 (92)	0.55 (85)		0.55 (86)	0.52 (35)	0.52 (56)	
rs5759195	G/G	0.12 (8)	0.13 (34)	0.9368	0.13 (15)	0.13 (27)	0.9931	0.14 (24)	0.12 (18)	0.2877	0.12 (18)	0.15 (10)	0.13 (14)	0.8769
	G/C	0.41 (28)	0.41 (107)		0.41 (47)	0.41 (88)		0.44 (77)	0.37 (58)		0.43 (67)	0.36 (24)	0.41 (44)	
	C/C	0.48 (33)	0.46 (120)		0.47 (54)	0.46 (99)		0.42 (74)	0.51 (79)		0.45 (70)	0.49 (33)	0.46 (50)	
rs762959	T/T	0.13 (9)	0.13 (34)	0.9998	0.14 (16)	0.13 (27)	0.9497	0.15 (26)	0.11 (17)	0.3826	0.12 (19)	0.15 (10)	0.13 (14)	0.9257
	T/C	0.42 (29)	0.42 (110)		0.41 (48)	0.43 (91)		0.43 (76)	0.41 (63)		0.43 (67)	0.37 (25)	0.44 (47)	
	C/C	0.45 (31)	0.45 (117)		0.45 (52)	0.45 (96)		0.42 (73)	0.48 (75)		0.45 (69)	0.48 (32)	0.44 (47)	
rs1261025	G/G	0.10 (7)	0.14 (37)	0.5349	0.13 (15)	0.14 (29)	0.9308	0.14 (24)	0.13 (20)	0.9637	0.13 (20)	0.10 (7)	0.16 (17)	0.4551
	G/A	0.45 (31)	0.47 (123)		0.46 (53)	0.47 (101)		0.47 (82)	0.46 (72)		0.47 (73)	0.55 (37)	0.41 (44)	
	A/A	0.45 (31)	0.39 (101)		0.41 (48)	0.39 (84)		0.39 (69)	0.41 (63)		0.40 (62)	0.34 (23)	0.44 (47)	
rs434651	A/A	0.28 (19)	0.22 (57)	0.3460	0.25 (29)	0.22 (47)	0.5163	0.22 (39)	0.24 (37)	0.3393	0.23 (35)	0.22 (15)	0.24 (26)	0.8155
	G/A	0.55 (38)	0.53 (139)		0.55 (64)	0.53 (113)		0.57 (100)	0.50 (77)		0.57 (88)	0.52 (35)	0.50 (54)	
	G/G	0.17 (12)	0.25 (65)		0.20 (23)	0.25 (54)		0.21 (36)	0.26 (41)		0.21 (32)	0.25 (17)	0.26 (28)	

* Numbers in squares are the numbers of genotypes of a certain type.

**Table 2:** SNPs whose genotypes significantly differed between the groups under specific genetic models of inheritance (chi-square test).

Model	Outcome *	SNP	df	χ^2^	*p*-Value
Additive	Study 2	rs858239	2	6.8	0.03
Additive	Study 1	rs858239	2	9.4	0.01
Additive	Study 4	rs907611	4	9.5	0.05
Additive	∆ = mRS14 − mRS1_4	rs907611	2	6.9	0.03
Additive	∆ = mRS14 − mRS1_1c	rs907611	2	8.7	0.01
Dominant	severity 5	rs34323745	2	8.7	0.01
Dominant	Study 1	rs858239	1	4.6	0.03
Dominant	Study 1	rs34323745	1	3.8	0.05
Dominant	∆ = mRS14 − mRS1_1b	rs494356	1	4.1	0.04
Dominant	∆ = mRS14 − mRS1_1c	rs907611	1	4.1	0.04
Recessive	severity 5	rs858239	2	6.9	0.03
Recessive	Study 2	rs858239	1	6	0.01
Recessive	Study 1	rs858239	1	6.4	0.01
Recessive	Study 4	rs907611	2	7.1	0.03
Recessive	∆ = mRS14 − mRS1_4	rs907611	1	5.1	0.02
Recessive	∆ = mRS14 − mRS1_1c	rs907611	1	5.7	0.02
Overdominant	severity 5	rs34323745	2	7.1	0.03
Overdominant	∆ = mRS14 − mRS1_1b	rs2089910	1	3.8	0.05

* Severity 5: mRS scores of 0–2 vs. scores of 3 vs. scores of 4–6; ∆ = mRS14 − mRS1_1a: ∆mRS < 0 vs. ∆mRS > 0; ∆ = mRS14 − mRS1_1b: ∆mRS < 0 vs. ∆mRS = 0; ∆ = mRS14 − mRS1_1c: ∆mRS > 0 vs. ∆mRS = 0; ∆ = mRS14 − mRS1_3: ∆mRS < 0 vs. (∆mRS > 0 + ∆mRS = 0); ∆ = mRS14 − mRS1_4: ∆mRS > 0 vs. (∆mRS < 0 + ∆mRS = 0).

**Table 3:** SNPs whose genotypes were significantly associated with IS outcome in logistic regression analysis.

Model	Outcome	Feature	Estimate	Std. Error	CI 2.5%	CI 97.5%	*p*-Value	OR	OR 2.5%	OR 97.5%
Additive	Study 1	rs858239GG	1.23	0.48	0.31	2.2	0.01	3.43	1.36	9
Additive	∆ = mRS14 − mRS1_3	rs494356TC	0.87	0.37	0.15	1.6	0.02	2.38	1.16	4.95
Additive	∆ = mRS14 − mRS1_4	rs907611GG	−1.15	0.48	−2.11	−0.2	0.02	0.32	0.12	0.82
Additive	∆ = mRS14 − mRS1_1b	rs494356TC	1.2	0.45	0.33	2.12	0.01	3.32	1.39	8.32
Additive	∆ = mRS14 − mRS1_1c	rs907611GG	1.35	0.61	0.18	2.58	0.03	3.84	1.19	13.24
Additive	Study 4	rs494356TC	0.87	0.34	0.22	1.54	0.01	2.4	1.25	4.66
Dominant	∆ = mRS14 − mRS1_3	rs494356	0.75	0.34	0.08	1.43	0.03	2.12	1.08	4.19
Dominant	∆ = mRS14 − mRS1_1b	rs494356	1.12	0.43	0.3	1.98	0.01	3.06	1.35	7.25
Dominant	Study 4	rs494356	0.81	0.32	0.19	1.44	0.01	2.24	1.21	4.21
Recessive	severity 5	rs858239	−0.61	0.28	−1.16	−0.06	0.03	0.55	0.31	0.94
Recessive	Study 2	rs858239	−0.79	0.3	−1.39	−0.2	0.01	0.45	0.25	0.82
Recessive	Study 1	rs858239	−0.89	0.34	−1.54	−0.22	0.01	0.41	0.21	0.8
Recessive	∆ = mRS14 − mRS1_4	rs907611	0.9	0.38	0.13	1.64	0.02	2.47	1.14	5.2
Recessive	∆ = mRS14 − mRS1_1b	rs494356	−1.11	0.42	−1.98	−0.3	0.01	0.33	0.14	0.74
Overdominant	∆ = mRS14 − mRS1_1b	rs494356	0.87	0.38	0.14	1.63	0.02	2.39	1.15	5.08
Overdominant	Study 4	rs494356	0.65	0.29	0.08	1.22	0.03	1.91	1.08	3.4

**Table 4:** Genomic characteristics of the polymorphisms studied, with primer and probe sequences and PCR conditions *.

SNP	chr	Position (GRCh38)	Alleles	Gene	Primer and Probe Sequences	Ta, °C
rs858239	7	23,246,696	G/A	GPNMB	5′-(FAM)TCAGGCAATGCCGC(BHQ1)-3′ 5′-(VIC) CAGGCAGTGCCGC(BHQ2)-3′ 5′-AGGAGTGAGTCATAAGC-3′ 5′-CCACCAAGAGCAACA-3′	57
rs34323745	7	23,272,449	C/A	GPNMB	5′-(FAM)CAGCAAAAACCTGTCTGAA(BHQ1)-3′ 5′-(VIC)CAGCAAAACCCTGTCTGA(BHQ2)-3′ 5′-GAGCCCAGAAGTCCAG-3′ 5′-ATCCCCACTGAATTAAAACC-3′	60
rs11267036	18	12,308,794	G/C	TUBB6	5′-(FAM)TGCTACTCACACGATGACTC(BHQ1)-3′ 5′-(VIC)TGCTACTCACAGGATGACTC(BHQ2)-3′ 5′-AGGCTACGTGGGAGA-3′ 5′-GCCGCGCTAAGAGG-3′	60
rs907611	11	1,852,842	G/A	LSP1	5′-(FAM)CCTGCGACCATCTTGG(BHQ1)-3′ 5′-(VIC) CTGCGGCCATCTTGG(BHQ2)-3′ 5′-CCCGAGCCATGAAGA-3′ 5′-GCTACAAGAGGAGAGGAA-3′	60
rs2089910	11	1,853,174	C/T	LSP1	5′-(FAM)TCCTCAGCACCCGG(BHQ1)-3′ 5′-(VIC)TCCTCGGCACCCG(BHQ2)-3′ 5′-CAGACTACAGGCTGATG-3′ 5′-AGACCCTTACCCCAG-3′	58
rs494356	11	117,201,254	T/C	TAGLN	5′-(FAM)AGTGGTCCTGCCCC(BHQ1)-3′ 5′-(VIC)AGTGGTCCCGCCC(BHQ2)-3′ 5′-CCAGTGCTCAGAGAAC-3′ 5′-CGGACTCATCGAAGTG-3′	60
rs664922	11	117,200,179	C/A	TAGLN	5′-(FAM)CTACATAAATGTGTGCCAT(BHQ1)-3′ 5′-(VIC)CTACATAAATTTGTGCCATC(BHQ2)-3′ 5′-GGTCTTTCCCAAAGG-3′ 5′-GGACAAACAGGAGTG-3′	55
rs5759195	22	43,152,613	G/C	TSPO	5′-(FAM)TGGCTCTGCTGTCCT(BHQ1)-3′ 5′-(VIC)TGGCTCTCCTGTCCT(BHQ2)-3′ 5′-GCCTACTGCCAGAAC-3′ 5′-CCAGGTGGAGACTCA-3′	55
rs762959	22	43,155,001	C/T	TSPO	5′-(FAM)TGGCACCCTATGCCC(BHQ1)-3′ 5′-(VIC)TGGCACCCCATGCC(BHQ2)-3′ 5′-CTGACATGGGTGCTCAC-3′ 5′-CCTTCATGCTGGAGGTTC-3′	58
rs1261025	1	13,593,698	A/G	PDPN	5′-(FAM) TCAGGGCGTGCTCA(BHQ1)-3′ 5′-(VIC)ATCAGGGCATGCTCA(BHQ2)-3′ 5′-AGGTCAGATGCAAAGG-3′ 5′-TCGGAAACTGAATGGAA-3′	57
rs434651	1	13,590,314	T/C	PDPN	5′-(FAM)TGCAAGCTGCAATCACA(BHQ1)-3′ 5′-(VIC)TGCAAGCTACAATCACAG(BHQ2)-3′ 5′-CAGTGCTGGGAGTAC-3′ 5′-GTTGCTTGTATGTTCTTTC-3′	58

* Ta—annealing temperature.

## Data Availability

All the data are presented in the manuscript and the Supplementary Materials.

## Supplementary Materials

The following are available online at https://www.mdpi.com/article/10.3390/ijms24076831/s1.

## References

[1] Katan M., Luft A. (2018). Global Burden of Stroke. Semin. Neurol..

[2] O’Donnell M.J., Chin S.L., Rangarajan S., Xavier D., Liu L., Zhang H., Rao-Melacini P., Zhang X., Pais P., Agapay S. (2016). Global and regional effects of potentially modifiable risk factors associated with acute stroke in 32 countries (INTERSTROKE): A case-control study. Lancet.

[3] Bevan S., Traylor M., Adib-Samii P., Malik R., Paul N.L.M., Jackson C., Farrall M., Rothwell P.M., Sudlow C., Dichgans M. (2012). Genetic heritability of ischemic stroke and the contribution of previously reported candidate gene and genomewide associations. Stroke.

[4] Mishra A., Malik R., Hachiya T., Jürgenson T., Namba S., Posner D.C., Kamanu F.K., Koido M., Le Grand Q., Shi M. (2022). Stroke genetics informs drug discovery and risk prediction across ancestries. Nature.

[5] Lindgren A., Maguire J. (2016). Stroke Recovery Genetics. Stroke.

[6] Alawieh A., Zhao J., Feng W. (2018). Factors affecting post-stroke motor recovery: Implications on neurotherapy after brain injury. Behav. Brain Res..

[7] Söderholm M., Pedersen A., Lorentzen E., Stanne T.M., Bevan S., Olsson M., Cole J.W., Fernandez-Cadenas I., Hankey G.J., Jimenez-Conde J. (2019). Genome-wide association meta-analysis of functional outcome after ischemic stroke. Neurology.

[8] Mola-Caminal M., Carrera C., Soriano-Tárraga C., Giralt-Steinhauer E., Díaz-Navarro R.M., Tur S., Jiménez C., Medina-Dols A., Cullell N., Torres-Aguila N.P. (2019). PATJ Low Frequency Variants Are Associated With Worse Ischemic Stroke Functional Outcome. Circ. Res..

[9] Ibanez L., Heitsch L., Carrera C., Farias F.H.G., Del Aguila J.L., Dhar R., Budde J., Bergmann K., Bradley J., Harari O. (2022). Multi-ancestry GWAS reveals excitotoxicity associated with outcome after ischaemic stroke. Brain.

[10] Gallagher M.D., Chen-Plotkin A.S. (2018). The Post-GWAS Era: From Association to Function. Am. J. Hum. Genet..

[11] Nicholls H.L., John C.R., Watson D.S., Munroe P.B., Barnes M.R., Cabrera C.P. (2020). Reaching the End-Game for GWAS: Machine Learning Approaches for the Prioritization of Complex Disease Loci. Front. Genet..

[12] Zheng Q., Ma Y., Chen S., Che Q., Chen D. (2020). The Integrated Landscape of Biological Candidate Causal Genes in Coronary Artery Disease. Front. Genet..

[13] Dergunova L.V., Filippenkov I.B., Stavchansky V.V., Denisova A.E., Yuzhakov V.V., Mozerov S.A., Gubsky L.V., Limborska S.A. (2018). Genome-wide transcriptome analysis using RNA-Seq reveals a large number of differentially expressed genes in a transient MCAO rat model. BMC Genom..

[14] Ma R., Xie Q., Li Y., Chen Z., Ren M., Chen H., Li H., Li J., Wang J. (2020). Animal models of cerebral ischemia: A review. Biomed. Pharmacother..

[15] Narayan S.K., Grace Cherian S., Babu Phaniti P., Babu Chidambaram S., Rachel Vasanthi A.H., Arumugam M. (2021). Preclinical animal studies in ischemic stroke: Challenges and some solutions. Anim. Model. Exp. Med..

[16] Wang Y., Cai Y. (2016). Obtaining Human Ischemic Stroke Gene Expression Biomarkers from Animal Models: A Cross-species Validation Study. Sci. Rep..

[17] Brubaker D.K., Lauffenburger D.A. (2020). Translating preclinical models to humans. Science.

[18] Khvorykh G., Khrunin A., Filippenkov I., Stavchansky V., Dergunova L., Limborska S. (2021). A Workflow for Selection of Single Nucleotide Polymorphic Markers for Studying of Genetics of Ischemic Stroke Outcomes. Genes.

[19] Khrunin A.V., Khvorykh G.V., Rozhkova A.V., Koltsova E.A., Petrova E.A., Kimelfeld E.I., Limborska S.A. (2021). Examination of Genetic Variants Revealed from a Rat Model of Brain Ischemia in Patients with Ischemic Stroke: A Pilot Study. Genes.

[20] Kongsawasdi S., Klaphajone J., Wivatvongvana P., Watcharasaksilp K. (2019). Prognostic Factors of Functional Outcome Assessed by Using the Modified Rankin Scale in Subacute Ischemic Stroke. J. Clin. Med. Res..

[21] Tsou P.S., Sawalha A.H. (2020). Glycoprotein nonmetastatic melanoma protein B: A key mediator and an emerging therapeutic target in autoimmune diseases. FASEB J..

[22] Huang J.J., Ma W.J., Yokoyama S. (2012). Expression and immunolocalization of Gpnmb, a glioma-associated glycoprotein, in normal and inflamed central nervous systems of adult rats. Brain Behav..

[23] Nakano Y., Suzuki Y., Takagi T., Kitashoji A., Ono Y., Tsuruma K., Yoshimura S., Shimazawa M., Iwama T., Hara H. (2014). Glycoprotein nonmetastatic melanoma protein B (GPNMB) as a novel neuroprotective factor in cerebral ischemia-reperfusion injury. Neuroscience.

[24] Neal M.L., Boyle A.M., Budge K.M., Safadi F.F., Richardson J.R. (2018). The glycoprotein GPNMB attenuates astrocyte inflammatory responses through the CD44 receptor. J. Neuroinflamm..

[25] Budge K.M., Neal M.L., Richardson J.R., Safadi F.F. (2020). Transgenic Overexpression of GPNMB Protects Against MPTP-Induced Neurodegeneration. Mol. Neurobiol..

[26] Tanaka H., Shimazawa M., Kimura M., Takata M., Tsuruma K., Yamada M., Takahashi H., Hozumi I., Niwa J.I., Iguchi Y. (2012). The potential of GPNMB as novel neuroprotective factor in amyotrophic lateral sclerosis. Sci. Rep..

[27] Hendrickx D.A.E., van Scheppingen J., van der Poel M., Bossers K., Schuurman K.G., van Eden C.G., Hol E.M., Hamann J., Huitinga I. (2017). Gene expression profiling of multiple sclerosis pathology identifies early patterns of demyelination surrounding chronic active lesions. Front. Immunol..

[28] Hüttenrauch M., Ogorek I., Klafki H., Otto M., Stadelmann C., Weggen S., Wiltfang J., Wirths O. (2018). Glycoprotein NMB: A novel Alzheimer’s disease associated marker expressed in a subset of activated microglia. Acta Neuropathol. Commun..

[29] Radecki D.Z., Wang A.R., Johnson A.S., Overman C.A., Thatcher M.M., Iyer G., Samanta J. (2021). Gpnmb inhibits oligodendrocyte differentiation of adult neural stem cells by amplifying TGFβ1 signaling. bioRxiv.

[30] Diaz-Ortiz M.E., Seo Y., Posavi M., Cordon M.C., Clark E., Jain N., Charan R., Gallagher M.D., Unger T.L., Amari N. (2022). GPNMB confers risk for Parkinson’s disease through interaction with α-synuclein. Science.

[31] Lind L., Zanetti D., Ingelsson M., Gustafsson S., Ärnlöv J., Assimes T.L. (2021). Large-scale plasma protein profiling of incident myocardial infarction, ischemic stroke, and heart failure. J. Am. Heart Assoc..

[32] Nalls M.A., Pankratz N., Lill C.M., Do C.B., Hernandez D.G., Saad M., Destefano A.L., Kara E., Bras J., Sharma M. (2014). Large-scale meta-analysis of genome-wide association data identifies six new risk loci for Parkinson’s disease. Nat. Genet..

[33] Murthy M.N., Blauwendraat C., Guelfi S., Hardy J., Lewis P.A., Trabzuni D. (2017). Increased brain expression of GPNMB is associated with genome wide significant risk for Parkinson’s disease on chromosome 7p15.3. Neurogenetics.

[34] Sasayama D., Hattori K., Ogawa S., Yokota Y., Matsumura R., Teraishi T., Hori H., Ota M., Yoshida S., Kunugi H. (2017). Genome-wide quantitative trait loci mapping of the human cerebrospinal fluid proteome. Hum. Mol. Genet..

[35] Jongstra-Bilen J., Jongstra J. (2006). Leukocyte-specific protein 1 (LSP1): A regulator of leukocyte emigration in inflammation. Immunol. Res..

[36] Hwang S.H., Jung S.H., Lee S., Choi S., Yoo S.A., Park J.H., Hwang D., Shim S.C., Sabbagh L., Kim K.J. (2015). Leukocyte-specific protein 1 regulates T-cell migration in rheumatoid arthritis. Proc. Natl. Acad. Sci. USA.

[37] Liu L., Cara D.C., Kaur J., Raharjo E., Mullaly S.C., Jongstra-Bilen J., Jongstra J., Kubes P. (2005). LSP1 is an endothelial gatekeeper of leukocyte transendothelial migration. J. Exp. Med..

[38] Petri B., Kaur J., Long E.M., Li H., Parsons S.A., Butz S., Phillipson M., Vestweber D., Patel K.D., Robbins S.M. (2011). Endothelial LSP1 is involved in endothelial dome formation, minimizing vascular permeability changes during neutrophil transmigration in vivo. Blood.

[39] Hossain M., Qadri S.M., Su Y., Liu L. (2013). ICAM-1-mediated leukocyte adhesion is critical for the activation of endothelial LSP1. Am. J. Physiol.—Cell Physiol..

[40] Hossain M., Qadri S.M., Xu N., Su Y., Cayabyab F.S., Heit B., Liu L. (2015). Endothelial LSP1 Modulates Extravascular Neutrophil Chemotaxis by Regulating Nonhematopoietic Vascular PECAM-1 Expression. J. Immunol..

[41] Hannigan M., Zhan L., Ai Y., Huang C. (2001). Leukocyte-specific gene 1 protein (LSP1) is involved in chemokine KC-activated cytoskeletal reorganization in murine neutrophils in vitro. J. Leukoc. Biol..

[42] Anderson C.A., Boucher G., Lees C.W., Franke A., D’Amato M., Taylor K.D., Lee J.C., Goyette P., Imielinski M., Latiano A. (2011). Meta-analysis identifies 29 additional ulcerative colitis risk loci, increasing the number of confirmed associations to 47. Nat. Genet..

[43] Le N.P.K., do Nascimento A.F., Schneberger D., Quach C.C., Zhang X., Aulakh G.K., Dawicki W., Liu L., Gordon J.R., Singh B. (2022). Deficiency of leukocyte-specific protein 1 (LSP1) alleviates asthmatic inflammation in a mouse model. Respir. Res..

[44] Ganesh S.K., Tragante V., Guo W., Guo Y., Lanktree M.B., Smith E.N., Johnson T., Castillo B.A., Barnard J., Baumert J. (2013). Loci influencing blood pressure identified using a cardiovascular gene-centric array. Hum. Mol. Genet..

[45] Wu Z., Hu Z., Gao Y., Xia Y., Zhang X., Jiang Z. (2023). A computational approach based on weighted gene co-expression network analysis for biomarkers analysis of Parkinson’s disease and construction of diagnostic model. Front. Comput. Neurosci..

[46] Han M., Dong L.H., Zheng B., Shi J.H., Wen J.K., Cheng Y. (2009). Smooth muscle 22 alpha maintains the differentiated phenotype of vascular smooth muscle cells by inducing filamentous actin bundling. Life Sci..

[47] Dong L.H., Wen J.K., Liu G., McNutt M.A., Miao S.B., Gao R., Zheng B., Zhang H., Han M. (2010). Blockade of the ras-extracellular signal-regulated kinase 1/2 pathway is involved in smooth muscle 22α-mediated suppression of vascular smooth muscle cell proliferation and neointima hyperplasia. Arterioscler. Thromb. Vasc. Biol..

[48] Dvorakova M., Nenutil R., Bouchal P. (2014). Transgelins, cytoskeletal proteins implicated in different aspects of cancer development. Expert Rev. Proteom..

[49] Sun Y., Zhao Z., Hou L., Xiao Y., Qin F., Yan J., Zhou J., Jing Z. (2017). The regulatory role of smooth muscle 22 on the proliferation of aortic smooth muscle cells participates in the development of aortic dissection. J. Vasc. Surg..

[50] Xia X., Shao T., Luo J., Zhang X., Bao M., Ye L., Cheng H. (2020). TAGLN expression decreases in the wall of unruptured intracranial aneurysms in Chinese population. Int. J. Clin. Exp. Med..

[51] Wang J., Kang Z., Liu Y., Li Z., Liu Y., Liu J. (2022). Identification of immune cell infiltration and diagnostic biomarkers in unstable atherosclerotic plaques by integrated bioinformatics analysis and machine learning. Front. Immunol..

[52] Chen R., Zhang F., Song L., Shu Y., Lin Y., Dong L., Nie X., Zhang D., Chen P., Han M. (2014). Transcriptome profiling reveals that the SM22α-regulated molecular pathways contribute to vascular pathology. J. Mol. Cell. Cardiol..

[53] Granata A., Kasioulis I., Serrano F., Cooper J.D., Traylor M., Sinha S., Markus H.S. (2022). The Histone Deacetylase 9 Stroke-Risk Variant Promotes Apoptosis and Inflammation in a Human iPSC-Derived Smooth Muscle Cells Model. Front. Cardiovasc. Med..

[54] Shu Y.N., Zhang F., Bi W., Dong L.H., Zhang D.D., Chen R., Lv P., Xie X.L., Lin Y.L., Xue Z.Y. (2015). SM22α inhibits vascular inflammation via stabilization of IκBα in vascular smooth muscle cells. J. Mol. Cell. Cardiol..

[55] Cao G., Xuan X., Hu J., Zhang R., Jin H., Dong H. (2022). How vascular smooth muscle cell phenotype switching contributes to vascular disease. Cell Commun. Signal..

[56] Liu X., Wang Q., Zhu R. (2021). Association of GWAS-susceptibility loci with ischemic stroke recurrence in a Han Chinese population. J. Gene Med..

[57] Ugorski M., Dziegiel P., Suchanski J. (2016). Podoplanin—A small glycoprotein with many faces. Am. J. Cancer Res..

[58] Wang Y., Peng D., Huang Y., Cao Y., Li H., Zhang X. (2022). Podoplanin: Its roles and functions in neurological diseases and brain cancers. Front. Pharmacol..

[59] Suzuki-Inoue K., Osada M., Ozaki Y. (2017). Physiologic and pathophysiologic roles of interaction between C-type lectin-like receptor 2 and podoplanin: Partners from in utero to adulthood. J. Thromb. Haemost..

[60] Zhang X., Zhang W., Wu X., Li H., Zhang C., Huang Z., Shi R., You T., Shi J., Cao Y. (2018). Prognostic Significance of Plasma CLEC-2 (C-Type Lectin-Like Receptor 2) in Patients With Acute Ischemic Stroke. Stroke.

[61] Wu X., Zhang W., Li H., You S., Shi J., Zhang C., Shi R., Huang Z., Cao Y., Zhang X. (2019). Plasma C-type lectin-like receptor 2 as a predictor of death and vascular events in patients with acute ischemic stroke. Eur. J. Neurol..

[62] Meng D., Ma X., Li H., Wu X., Cao Y., Miao Z., Zhang X. (2021). A Role of the Podoplanin-CLEC-2 Axis in Promoting Inflammatory Response After Ischemic Stroke in Mice. Neurotox. Res..

[63] Qian S., Qian L., Yang Y., Cui J., Zhao Y. (2022). Podoplanin neutralization reduces thrombo-inflammation in experimental ischemic stroke by inhibiting interferon/caspase-1/GSDMD in microglia. Ann. Transl. Med..

[64] De Meyer S.F., Denorme F., Langhauser F., Geuss E., Fluri F., Kleinschnitz C. (2016). Thromboinflammation in Stroke Brain Damage. Stroke.

[65] Hatakeyama K., Kaneko M.K., Kato Y., Ishikawa T., Nishihira K., Tsujimoto Y., Shibata Y., Ozaki Y., Asada Y. (2012). Podoplanin expression in advanced atherosclerotic lesions of human aortas. Thromb. Res..

[66] Furukoji E., Yamashita A., Nakamura K., Hirai T., Asada Y. (2019). Podoplanin expression on endothelial cells promotes superficial erosive injury and thrombus formation in rat carotid artery: Implications for plaque erosion. Thromb. Res..

[67] Shetova I.M., Timofeev D.I., Shamalov N.A., Bondarenko E.A., Slominskiĭ P.A., Limborskaia S.A., Skvortsova V.I. (2012). The association between the DNA marker rs1842993 and risk for cardioembolic stroke in the Slavic population. Zhurnal Nevrol. i psikhiatrii Im. S.S. Korsakova.

[68] Brott T., Adams H.P., Olinger C.P., Marler J.R., Barsan W.G., Biller J., Spilker J., Holleran R., Eberle R., Hertzberg V. (1989). Measurements of acute cerebral infarction: A clinical examination scale. Stroke.

[69] van Swieten J.C., Koudstaal P.J., Visser M.C., Schouten H.J., van Gijn J. (1988). Interobserver agreement for the assessment of handicap in stroke patients. Stroke.

[70] Milligan B.G., Hoelzel A. (1998). Total DNA isolation. Molecular Genetic Analysis of Populations.

[71] Ward L.D., Kellis M. (2016). HaploReg v4: Systematic mining of putative causal variants, cell types, regulators and target genes for human complex traits and disease. Nucleic Acids Res..

[72] Schwarz J.M., Hombach D., Köhler S., Cooper D.N., Schuelke M., Seelow D. (2019). RegulationSpotter: Annotation and interpretation of extratranscriptic DNA variants. Nucleic Acids Res..

[73] Venables W.N., Ripley B.D. (2002). Modern Applied Statistics with S.

[74] Barrett J.C., Fry B., Maller J., Daly M.J. (2005). Haploview: Analysis and visualization of LD and haplotype maps. Bioinformatics.

[75] Zeileis A., Kleiber C., Jackman S. (2008). Regression Models for Count Data in R. J. Stat. Softw..

[76] Lesnoff M., Lancelot R. (2010). aod: Analysis of Overdispersed Data 2010.

