# Supplementary material for: The Study of the Association of Polymorphisms in LSP1, GPNMB, PDPN, TAGLN, TSPO, and TUBB6 Genes with the Risk and Outcome of Ischemic Stroke in the Russian Population
Source: Int J Mol Sci. 2023 Apr 6;24(7):6831. doi: 10.3390/ijms24076831 (PMC10095190; doi:10.3390/ijms24076831)
Supplement: Supplementary file 1 [file ijms-24-06831-s001.zip › Table S2.pdf]

Supplementary Table S2. Characteristics of genetic models of inheritance tested in the study

SNP	a1*	a2	Additive	Dominant<br>1, 0	Recessive<br>1, 0	Overdominant<br>1, 0
rs858239	A	G	AA, AG, GG	AA/AG, GG	AA, AG/GG	AG, AA/GG
rs34323745	A	C	AA, AC, CC	AA/AC, CC	AA, AC/CC	AC, AA/CC
rs11267036	G	C	GG, GC, CC	GG/GC, CC	GG, GC/CC	GC, GG/CC
rs907611	A	G	AA, AG, GG	AA/AG, GG	AA, AG/GG	AG, AA/GG
rs2089910	A	G	AA, AG, GG	AA/AG, GG	AA, AG/GG	AG, AA/GG
rs494356	T	C	TT, TC, CC	TT/TC, CC	TT, TC/CC	TC, TT/CC
rs664922	G	T	GG, GT, TT	GG/GT, TT	GG, GT/TT	GT, GG/TT
rs5759195	G	C	GG, GC, CC	GG/GC, CC	GG, GC/CC	GC, GG/CC
rs762959	T	C	TT, TC, CC	TT/TC, CC	TT, TC/CC	TC, TT/CC
rs1261025	G	A	GG, GA, AA	GG/GA, AA	GG, GA/AA	GA, GG/AA
rs434651	A	G	AA, AG, GG	AA/AG, GG	AA, AG/GG	AG, AA/GG

*a1 – minor allele
